# Phenotypical and functional characteristics of mesenchymal stem cells from bone marrow: comparison of culture using different media supplemented with human platelet lysate or fetal bovine serum

**DOI:** 10.1186/scrt97

**Published:** 2012-02-14

**Authors:** Nesrine Ben Azouna, Faouzi Jenhani, Zohra Regaya, Lamia Berraeis, Tarek Ben Othman, Elfi Ducrocq, Jorge Domenech

**Affiliations:** 1 Laboratory of Hematopoiesis, UPRES-EA3855, Faculty of Medicine, University François Rabelais, 10 boulevard Tonnellé, 37032 Tours Cedex, France; 2 Immunology Research Unit, Faculty of Pharmacy, rue Ibn Sina, 5000 Monastir, Tunisia; 3 Cell Immunology, Cytometry and Cell Therapy Laboratories, National Blood Centre of Tunisia, Bab Sadoun, 1002 Tunis, Tunisia; 4 National Centre of Bone Marrow Graft (CNGMO), Bab Sadoun, 1002 Tunis, Tunisia

## Abstract

**Introduction:**

Mesenchymal stem cells (MSCs) are multipotent cells able to differentiate into several mesenchymal lineages, classically derived from bone marrow (BM) but potentially from umbilical cord blood (UCB). Although they are becoming a good tool for regenerative medicine, they usually need to be expanded in fetal bovine serum (FBS)-supplemented media. Human platelet lysate (HPL) has recently been proposed as substitute for safety reasons, but it is not yet clear how this supplement influences the properties of expanded MSCs.

**Methods:**

In the present study, we compared the effect of various media combining autologous HPL with or without FBS on phenotypic, proliferative and functional (differentiation, cytokine secretion profile) characteristics of human BM-derived MSCs.

**Results:**

Despite less expression of adipogenic and osteogenic markers, MSCs cultured in HPL-supplemented media fully differentiated along osteoblastic, adipogenic, chondrogenic and vascular smooth muscle lineages. The analyses of particular specific proteins expressed during osteogenic differentiation (calcium-sensing receptor (CaSR) and parathormone receptor (PTHR)) showed their decrease at D0 before any induction for MSC cultured with HPL mostly at high percentage (10%HPL). The cytokine dosage showed a clear increase of proliferation capacity and interleukin (IL)-6 and IL-8 secretion.

**Conclusions:**

This study shows that MSCs can be expanded in media supplemented with HPL that can totally replace FBS. HPL-supplemented media not only preserves their phenotype as well as their differentiation capacity, but also shortens culture time by increasing their growth rate.

## Introduction

Mesenchymal stem cells (MSCs) represent a rare population of multipotent progenitors, initially described in bone marrow (BM), giving rise to adipocytes, osteoblasts, chondrocytes, and vascular smooth muscle (VSM)-like hematopoietic supportive stromal cells [1,2]. These cells are capable of multilineage differentiation from a single cell [3,4] and *in vivo *functional reconstitution of injured tissues in preclinical [5] as well in clinical [6] settings. Thereafter, MSCs have been described in virtually all post-natal organs or tissues [7] but also in fetal adnexa, including both umbilical cord blood [8,9] and placenta [7,10,11]. Today, BM remains the principal source of MSCs in studies investigating their potential use in cell therapy, in spite of significant declines in cell proliferation and differentiation capacity with advancing age [12].

MSCs display several common surface antigens, including CD90 (Thy-1), CD166 (SB10/ALCAM), CD73 (SH3), and CD105 (SH2 and endoglin), and lack hematopoietic (such as CD45 or CD34) and endothelial (such as CD31) markers and HLA-DR. However, significant heterogeneity in the morphology, proliferation, and differentiation potentials of MSCs has also been observed [13]. Fetal bovine serum (FBS)-based medium is the conventional medium for isolation and expansion of MSCs and has been used in several clinical trials [6]. However, concerns over the safety of FBS-based culture media have been raised because they can trigger adverse responses [14,15]. Accordingly, to culture MSCs, some trials have used media containing animal serum-free substitutes with combinations of growth factors [16]. An alternative is to supplement media with human blood-derived platelet lysates or sera from autologous or allogenic donors [17-21] to create culture conditions that more closely mimic the human environment. However, it is not yet clear how these culture conditions influence the phenotype and function of the expanded MSC populations.

In the present study, we have tested different media, including combinations of autologous human platelet lysate (HPL) with or without FBS, in order to compare phenotypic, proliferative, and functional characteristics together with the cytokine secretion profile of human MSCs derived from BM. We focused particularly on the mesenchymal differentiation ability of MSCs expanded in these different conditions.

## Materials and methods

### Mesenchymal stem cell expansion cultures from bone marrow

BM cells (*n *= 13) were isolated from patients with normal BMs harvested from donors at the Bone Marrow Graft National Center of Tunisia (Tarek, Ben Othman, Tunis) or during orthopedic surgery (Philippe Rosset, Orthopedics Department, Hôpital Trousseau, Tours). The mean age of the donors was 33 ± 2 years (range of 16 to 41 years). Informed consent was obtained from all of the patients (a written consent was provided by the patients from Tours) in accordance with the national ethical guidelines of each country.

Mononuclear cells (MNCs) were isolated from each BM sample by loading onto Ficoll Hypaque solution (d = 1.077). After centrifugation at 800*g *for 20 minutes at room temperature, the MNC layer was removed from the interphase and washed twice with phosphate-buffered saline (PBS). Then they were seeded into uncoated T25 or T75 flasks (Becton, Dickinson and Company, Franklin Lakes, NJ, USA) at a cell concentration of 1 × 10^5 ^cells per square centimeter for BM cells. Cells were cultured in basic growth medium (BGM) consisting of alpha-minimum essential medium (α-MEM) (Invitrogen Corporation, Carlsbad, CA, USA) with 100 U/mL penicillin, 0.1 mg/mL streptomycin (Invitrogen), 2 mM L-glutamine (Invitrogen), 0.025 mg/mL amphotericin B (Invitrogen) with various supplements to obtain the four following expansion culture media: (M1) BGM with 10% (vol/vol) of prescreened FBS (HyClone, Thermo Fisher Scientific Inc., Waltham, MA, USA) and 1 ng/mL fibroblast growth factor 2 (FGF2) (AbCys, Paris, France), (M2) BGM with 10% FBS and 5% (vol/vol) HPL, (M3) BGM with 10% HPL, and (M4) BGM with 5% HPL. Cultures were maintained in a humidified atmosphere with 5% CO_2 _at 37°C and the medium had been changed twice weekly thereafter. On reaching 60% to 80% confluence, the adherent cells were detached after treatment with 0.05% (vol/vol) trypsin/1 mM ethylenediaminetetraacetic acid (EDTA) solution (Invitrogen) and reseeded at 10^3 ^cells per square centimeter. All of the studies, except for cell proliferation and CFU-F assays, had been performed after two passages (P2).

### Human platelet lysate preparation

HPLs were prepared in accordance with a method previously described [19]. Briefly, they were obtained from different platelet apheresis collections performed at the National Blood Transfusion Center of Tunis (Tunisia). HPLs from at least 10 donors were pooled and, after elimination of remaining platelet bodies by centrifugation (1,400*g *for 20 minutes), were frozen at -80°C to be subsequently used as FBS or growth factor substitute or both.

### Phenotypic analysis of mesenchymal stem cells by flow cytometry

The phenotypic analysis was performed after P2 by incubation of the adherent cells with monoclonal antibodies. Cultures were washed twice with PBS, and cells were subsequently detached by enzymatic treatment by using 0.05% trypsin/EDTA solution (Invitrogen) for 5 minutes at 37°C. Cells were washed and adjusted to 1 × 10^6^/mL in PBS. Cell suspensions (100 mL) were incubated with 5 μL of fluorescein isothiocyanate (FITC)-, phycoerythrin (PE)-, or peridinin chlorophyll protein (PerCP)-conjugated IgG1 monoclonal antibodies (Becton, Dickinson and Company) for 30 minutes at room temperature in the dark. They were CD45-PerCP, CD14-PE, CD31-PE, CD146-FITC, CD34-PE, CD90-FITC or CD90-PE, CD105-PE, CD106-PE, and CD133-PE (BD Biosciences, San Jose, CA, USA). Nonspecific background was evaluated by parallel staining with isotype-matched IgG1-FITC, IgG1-PerCP, and IgG1-PE. Cells were fixed with 4% paraformaldehyde and analyzed by using a Becton, Dickinson and Company flow cytometer (FACScalibur^™ ^or FACS Canto II^™^).

### Mesenchymal differentiation capacity of mesenchymal stem cells

#### Osteogenic induction

At 50% confluence, the cells were cultured for 14 days in Dulbecco's modified Eagle's medium-high glucose (DMEM-HG) (with 4.5 g/L glucose) containing 2% FBS, 0.1 μM dexamethasone (Sigma-Aldrich, St. Louis, MO, USA), 2 mM β-glycerolphosphate (Sigma-Aldrich), and 100 μM ascorbate-2-phosphate (Sigma-Aldrich) with medium changes every 3 days. After 2 weeks of induction, the cells were stained in accordance with von Kossa and Alizarin red methods to detect the presence of calcium deposition into osteocytes.

#### Adipogenic induction

The adipogenic induction medium consisted of DMEM-LG (with 1 g/L glucose) supplemented with 20% FBS, 1 μM/L dexamethasone, 0.5 mmol/L isobutylmethylxanthine (Invitrogen), and 60 μmol/L indomethacin (Sigma-Aldrich). Adipogenic differentiation was evaluated after 2 weeks of induction by the cellular accumulation of neutral lipid vacuoles that were stained with Nile red (Sigma-Aldrich) and observed by fluorescent microscopy.

#### Chondrogenic induction

Chondrogenic differentiation was obtained in micropellets (3 × 10^5 ^cells per pellet) incubated in tubes at 37°C for 21 days in 500 μL of chondrogenic medium consisting of DMEM-HG with 0.1 μM dexamethasone (Sigma-Aldrich), 1 mM sodium pyruvate, 170 μM ascorbic acid-2-phosphate (Sigma-Aldrich), 350 μM proline (Sigma-Aldrich), 1X insulin-transferrin-selenium (Cambrex, East Rutherford, NJ, USA), and 10 ng/mL transforming growth factor-beta-1 (TGF-β1) (AbCys). Medium was changed every 4 days. Chondrogenic differentiation was evaluated after 21 days of induction. The presence of glycosaminoglycans in cell pellets was revealed by Alcian blue and Toluidine blue staining. On the 21st day, pellets were fixed for 48 hours in 4% paraformaldehyde and embedded in paraffin. Sections (4 μm thick) were rehydrated in 70%, 90%, and 100% xylene and ethanol. For Alcian blue staining, slides were incubated for 10 minutes in a 1:1 mixture of 3% (vol/vol) Alcian blue 8GX (Sigma-Aldrich) solution in distilled water and 3% (vol/vol) methanol. For Toluidine blue staining, slides were incubated for 10 minutes in a 1% Toluidine blue (Sigma-Aldrich) (vol/vol) solution in 70% ethanol.

#### Vascular smooth muscle induction

VSM differentiation was obtained in McCoy's 5A medium supplemented with 12.5% FBS, 12.5% horse serum (Invitrogen), 2 mM L-glutamine, 0.8 mM L-serine, 0.15 mM L-asparagine, 1 mM sodium pyruvate, 5 mM sodium bicarbonate, 1 μM hydrocortisone and 0.25 μg/mL amphotericin B. Medium was changed every 4 days. VSM differentiation was evaluated after 3 weeks of induction. MSCs were fixed by using 4% paraformaldehyde and incubated with primary antibodies against rat alpha smooth muscle actin (ASMA) (clone 1A4, mouse anti-human, -mouse, -rat; Sigma-Aldrich) at 4°C for 12 hours. The antigen-antibody reaction was detected by using a specific anti-mouse secondary antibody coupled to FITC (Alexa Fluor 488; Invitrogen) dyes, and the reaction was visualized by fluorescent microscopy.

### Mesenchymal stem cell expression of differentiation markers by RT-PCR and Western blotting

For reverse transcription-polymerase chain reaction (RT-PCR), total RNA was extracted from MSCs at 90% confluence by using TRIZOL reagent (Invitrogen) in accordance with the instructions of the manufacturer. Reverse transcription was carried out by using PrimeScript^™^RTase (Takara, Kyoto, Japan), and the cDNA fragments were amplified by using RNase Inhibitor (Takara). After denaturation at 65°C for 5 minutes, amplification was carried out by 30 cycles at 30°C for 10 minutes, 42°C for 60 minutes, and 95°C for 5 minutes. Primers used are shown in Table 1. The expression of the following genes was studied: alkaline phosphatase (*ALP*) nd Runt-related transcription factor 2 (*RUNX2*) for osteogenic pathway, peroxisome proliferator-activated receptor-gamma (*PPARγ*) and lipoprotein lipase (*LPL*) for adipogenic pathway, and *ASMA *for VSM differentiation. Glyceraldehyde 3-phosphate dehydrogenase (*GAPDH*) gene expression was used as a control. Thermocycling was performed with a gradient thermocycler (Takara).

**Table 1:** Sequences of the primers used in reverse transcription-polymerase chain reaction study

Construct	Forward sequence	Reverse sequence
*GAPDH*	5'- AATCCCATCACCATCTTCCAGG-3'	5'-AGAGGCAGGGATGATGTTCTGG-3'
*ALP*	5'-CTGGACCTCGTTGACACCTG-3'	5'-GACATTCTCTCGTTCACCGC-3'
*RUNX2*	5'-AACTTCCTGTGCTCGGTGCTG-3'	5'-GGGGAGGATTTGTGAAGACGG-3'
*LPL*	5'-AAAGCCCTGCTCGTGCTGAC-3'	5'-TAAACCGGGCCACATCCTGT-3'
PPARγ	5'-GGAGAAGCTGTTGGCGGAGA-3'	5'-TCAAGGAGGCCAGCATTGTG-3'
ASMA	5'-TCATGATGCTGTTGTAGGTGGT-3'	5'-CTGTTCCAGCCATCCTTCAT-3'

*ALP*, alkaline phosphatase; *ASMA*, alpha smooth muscle actin; *GAPDH*, glyceraldehyde 3-phosphate dehydrogenase; *LPL*, lipoprotein lipase; PPARγ, peroxisome proliferator-activated receptor-gamma; *RUNX2*, Runt-related transcription factor 2.

For Western blotting, MSCs were harvested at 90% confluence. Cell pellets were resuspended in lysis buffer (8 M urea, 2 M thiourea, 2% CHAPS, 1% NP-40, 2 mM TBP, 16 Protease Inhibitor Mix, 16 Nuclease Mix, 1 mM PMSF, and 2% IPG buffer) and put on ice for 45 minutes. They were then centrifuged at 14,000*g *for 15 minutes at 4°C. The supernatant was collected and stored at -80°C. The protein concentration of samples was determined by means of Bradford's method (Bio-Rad Laboratories, Inc., Hercules, CA, USA). Equal amounts of cell extracts and of conditioned media were separated by SDS-PAGE (12%) and transferred to polyvinylidene difluoride (PVDF) membranes. Membranes were blocked with 5% skim milk for an hour and left to react with anti-human β-actin (clone AC-15, mouse IgG1; Sigma-Aldrich) as a positive control, anti-human leptin (monoclonal; R&D Systems, Inc., Minneapolis, MN, USA, reference number 398), anti-human calcium-sensing receptor (CaSR) (monoclonal; AbCam, Cambridge, UK, reference number ab 3513), anti-human parathormone receptor (PTHR) (mouse monoclonal anti-human; Novocastra, part of Leica Microsystems, Wetzlar, Germany), anti-human ASMA (clone 1A4, mouse anti-human, -mouse, -rat; Sigma-Aldrich), and anti-human transgelin (SM22α) (mouse monoclonal anti-human; Novocastra). Antibody binding was revealed by incubation with the corresponding HRP-linked IgG (horseradish peroxydase; Bio-Rad Laboratories, Inc.) and the ECL+ Western blotting detection kit (Amersham Biosciences, now part of GE Healthcare, Little Chalfont, Buckinghamshire, UK). Chemiluminescence was detected and measured with a Chemi-Smart 2000^™ ^Imager (Vilber Lourmat, Marne-la-Vallée, France).

### Clonogenic and proliferative capacities of mesenchymal stem cells

#### Colony-forming unit-fibroblast assays

CFU-F assays were performed to evaluate the clonal expansion capacity of the MSCs initially cultured in the six different media. For the optimal counting of colonies, MNCs derived from BM were seeded at 10^5 ^cells per square centimeter in T25 flasks in duplicate. Flasks were incubated in a humidified atmosphere with 5% CO_2 _at 37°C. The medium was completely renewed every 3 days. Cell clusters containing more than 50 cells were scored on day 10 as CFU-F colonies by using an inverted microscope. Cloning efficiency was considered as CFU-F counts per 10^6 ^MNCs plated.

#### Population doubling time

To test the role of culture conditions on MSC expansion, MSCs were cultured in each of the six expansion media for two passages (P2) before analysis. Population doubling times (PDTs) were calculated between P1 and P2 as t/n, where t is the duration of culture in days and n is the number of population doublings calculated by using the formula *n *= (log N_h _- log N_i_)/log 2, where N_h _is the number of cells harvested at the time of counting at P2 and N_i _is the number of cells initially plated at P1).

### Cytokine secretion capacity of mesenchymal stem cells

Cytokines were detected in supernatants of MSCs cultured in different expansion media and were collected at P2. Concentrations of IL-6, IL-8, vascular endothelial growth factor (VEGF), FGF2, granulocyte colony-stimulating factor (G-CSF), granulocyte-macrophage colony-stimulating factor (GM-CSF), and RANTES (regulated on activation normal T cell expressed and secreted) were simultaneously evaluated by using a commercially available multiplex bead-based immunoassay (Bio-Plex^™ ^cytokine assay system; BD Biosciences).

### Statistical analysis

Statistical analysis was performed by using the STATGRAPHICS Centurion XVI program version 16.0.08 (StatPoint Technologies Inc., Warrenton, VA, USA). A one-way analysis of variance and Newman-Keuls multiple range test were carried out to compare value distributions. Significant difference between group values was considered when *P *value was less than 0.05. Indicated values are mean ± standard error mean.

## Results

### Phenotypic characteristics of mesenchymal stem cells cultured in various expansion media

#### Morphological analysis

**Figure 1:**
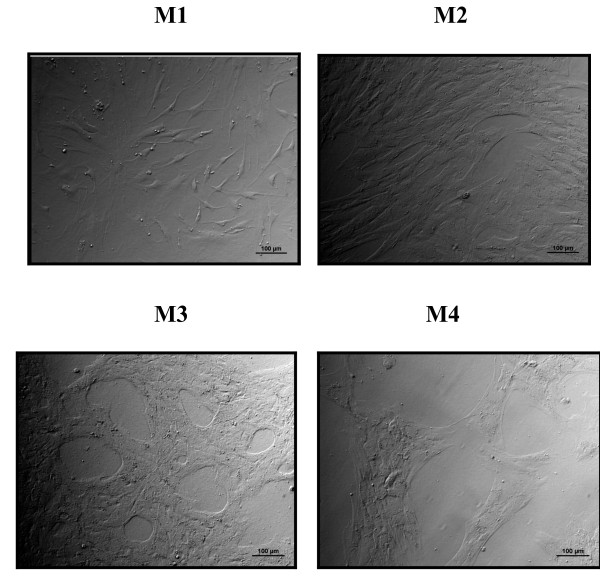
**Morphological characterization of bone marrow (BM)-derived mesenchymal stem cells (MSCs) cultured in different media at passage 2 (P2)**. The media were M1 (10% FBS + FGF2), M2 (10% FBS + 5% HPL), M3 (10% HPL), and M4 (5% HPL). Bar represents 100 μm. FBS, fetal bovine serum; FGF2, fibroblast growth factor 2; HPL, human platelet lysate. Representative results from 13 experiments are shown.

Adherent cell populations from the MNC fraction of human BM samples were generated by expansion culture by using four different media: (M1) BGM with 10% FBS and 1 ng/mL FGF2, (M2) BGM with 10% FBS and 5% HPL, (M3) BGM with 10% HPL, and (M4) BGM with 5% HPL. After 2 weeks of culture, an adherent and stable cell layer was obtained from BM-derived MNCs with all media (*n *= 13). Figure 1 shows a particular morphology of MSCs cultured with HPL (M3 and M4). In fact, the layer of MSCs appears with many spaces between the cells in comparison with standard medium (M1), in which we have one layer of very confluent MSCs (Figure 1).

#### Immunophenotype analyzed by flow cytometry

**Figure 2:**
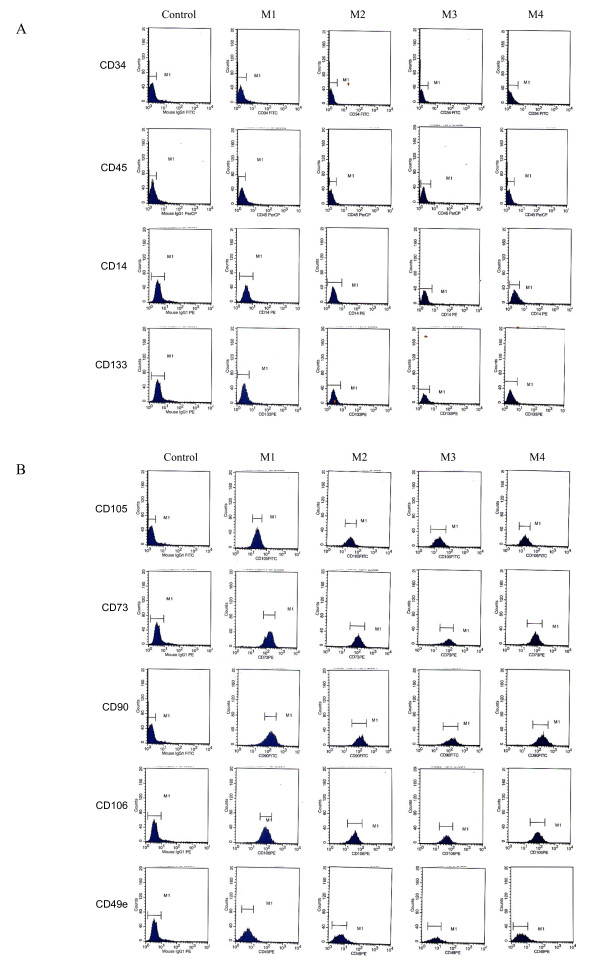
**Representative flow cytometry analysis of BM-derived MSCs cultured in different expansion media**. Comparison of membrane antigen expression of MSCs cultured at passage 2 (P2) in four different media: M1 (10% FBS + FGF2) (*n *= 4), M2 (10% FBS + 5% HPL) (*n *= 5), M3 (10% HPL) (*n *= 6), and M4 (5% HPL) (*n *= 5). The histogram plots represent flow cytometry analysis of the cells with directly labeled monoclonal antibodies (histograms colored inside) or exposed to isotype-matched non-immune directly labeled immunoglobulins (IgG1FITC, IgG1PE, and IgG1PerCP). MSCs were negative for hematopoietic markers CD45, CD34, CD133, and CD14 (A) but were positive for CD90, CD73, CD105, and CD106 and weakly positive for CD49e (B). (A) Hematopoietic and immaturity markers. (B) Stromal cell markers.

MSCs from different BM samples were characterized by flow cytometry with a panel of six markers at P2 after culture in media containing HPL (M2, M3, M4) or not containing HPL (M1). All BM derived-MSCs were negative for hematopoietic markers CD34, CD14, and CD45 (Figure 2A) and were consistently positive for the MSC markers CD106, CD73, CD90, and CD105, regardless of the expansion medium used (Figure 2B). For immaturity markers, expression of CD49a was low and CD133 was undetectable without any influence of medium type. Our data did not show any statistical difference between the four culture conditions.

### Mesenchymal differentiation capacity of mesenchymal stem cells assessed by specific staining in different culture conditions

**Figure 3:**
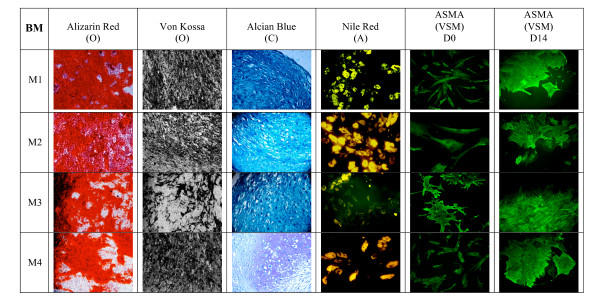
**Osteogenic, chondrogenic, adipogenic, and vascular smooth muscular differentiation capacity assessed by staining of BM-derived MSCs cultured in different expansion media**. The media are M1 (10% FBS + FGF2), M2 (10% FBS + 5% HPL), M3 (10% HPL), and M4 (5% HPL). Results are of one representative experiment. A, adipogenic differentiation; ASMA, alpha smooth muscle actin; C, chondrogenic differentiation; D0, before differentiation; D14, after 14 days of differentiation; O, osteogenic differentiation; VSM, vascular smooth muscle.

The influence of HPL in expansion media on further differentiation potential of MSCs toward osteogenic, adipogenic, chondrogenic, and VSM lineages after appropriate induction was investigated at the second passage (P2) and specific staining (Figure 3). BM cells grown in HPL or FBS deposited an extensive mineralized matrix when cultured for 2 weeks in osteogenic medium, as demonstrated by strong Alizarin red and von Kossa staining. These cells also efficiently differentiated into the adipogenic lineage, as indicated by Nile red staining of lipid droplets in the cytoplasm following culture in adipogenic medium except when a high concentration (10%) of HPL was used (M3). After chondrogenic differentiation for 3 weeks, MSCs displayed a typical chondroblast shape, as indicated by the deposition of specific glycosaminoglycans seen after Alcian blue and O Safranin staining, irrespectively of the expansion media used previously (M1, M2, M3, and M4).

### Osteogenic, adipogenic, and vascular smooth muscle gene expression assessed by RT-PCR analysis in mesenchymal stem cells

**Figure 4:**
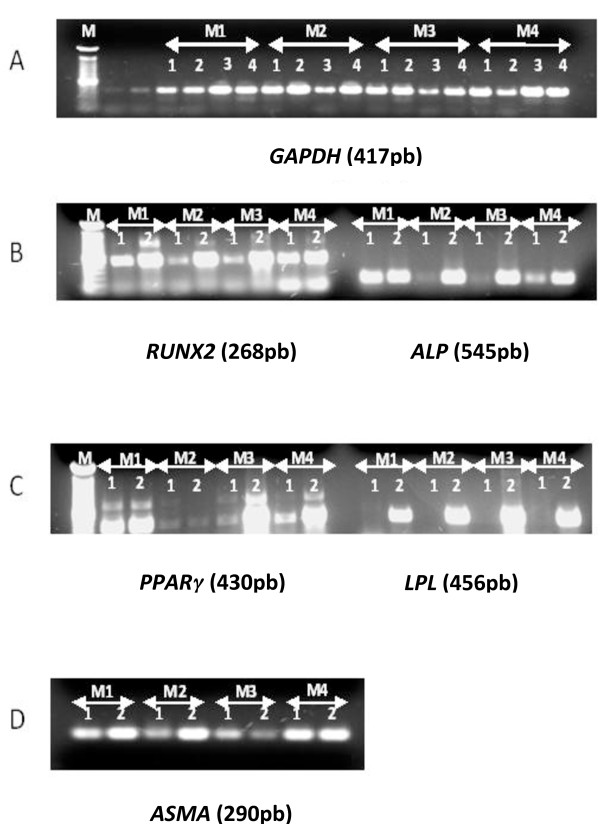
**mRNA expression by BM-derived MSCs cultured in different expansion media**. Reverse transcription-polymerase chain reaction (RT-PCR) for positive control *GAPDH *(A), osteogenic (B), adipogenic (C), and vascular smooth muscle (D) gene expression of BM-derived MSCs cultured in four media: M1 (10% FBS + FGF2), M2 (10% FBS + 5% HPL), M3 (10% HPL), and M4 (5% HPL). Total RNA was extracted from undifferentiated and differentiated MSCs previously cultured in four expansion media (M1, M2, M3, and M4). The cDNA obtained was used for PCR assays to evaluate the expression of the different genes studied. One representative experiment out of four experiments is shown. For GAPDH gene expression (A): 1, at D0 before any differentiation; 2, after osteogenic differentiation (D14); 3, after adipogenic differentiation (D14); 4, after vascular smooth muscle differentiation (D14). For osteogenic (*RUNX2 *and *ALP*) (B), adipogenic (*PPARγ *and *LPL*) (C), and vascular smooth muscle (*ASMA*) (D) gene expression: 1, D0 before any differentiation; 2, after differentiation induction (D14). *ALP*, alkaline phosphatase; *ASMA*, alpha-smooth muscle actin; *GAPDH*, glyceraldehyde 3-phosphate dehydrogenase; *LPL*, lipoprotein lipase; *PPARγ*, peroxisome proliferator-activated receptor-γ; *RUNX2*, runt-related transcription factor 2.

Gene expression was analyzed by RT-PCR after MSC amplification (P1) in HPL- and FBS-supplemented media before (day 0, or D0) and after differentiation (day 14, or D14) from four different MSCs. We tested the expression of two osteogenic (O)-specific genes (*ALP *and *RUNX2*), two adipogenic (A)-specific genes (*PPARγ *and *LPL*), and one VSM gene (*ASMA*) before differentiation. mRNA expressions of *ALP, RUNX2, PPRγ*, and *ASMA *were decreased in M2 (10% FBS + 5% HPL) and M3 (10% HPL) media in comparison with standard M1 medium (10% FBS + 1 ng/mL FGF2) or M4 medium containing only 5% HPL (Figure 4A). At D0, HPL seems to decrease the osteogenic markers. Concerning the adipogenic markers, *PPARγ *decreased at D0 when the HPL was added to the culture medium (M2, M3, and M4) in comparison with standard medium (M1). Also, in M2 (10% FBS + 5% HPL), we showed a decrease of *PPARγ *after adipogenic induction at D14.

### Osteogenic, adipogenic, and vascular smooth muscular protein expression assessed by Western blotting in mesenchymal stem cells

**Figure 5:**
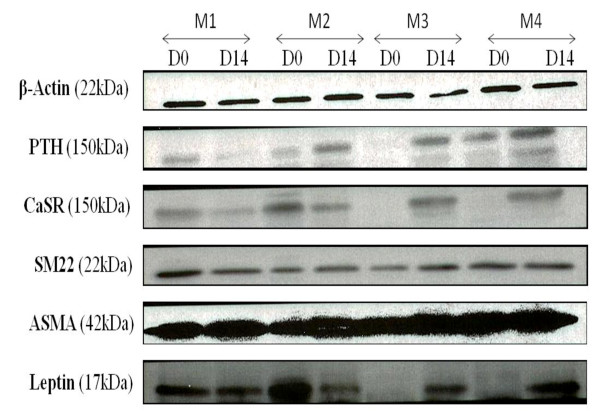
**Western blot analysis showing differentiation markers of osteogenic, vascular smooth muscle, and adipogenic lineages expressed by BM-derived MSCs cultured in different expansion media**. The media are M1 (10% FBS + FGF2), M2 (10% FBS + 5% HPL), M3 (10% HPL), and M4 (5% HPL). The experiments were performed in triplicate, and one representative blot is shown here. Beta-actin expression was used as positive control. Osteogenic proteins studied are calcium-sensing receptor (CaSR) and parathormone receptor (PTHR). Adipogenic protein studied is Leptin. Vascular smooth muscle proteins studied are smooth muscle 22alpha (SM22α) and alpha-smooth muscle actin (ASMA). D0, day 0 (before induction); D14, day 14 (after induction).

Proteins specific of osteogenic, VSM, and adipogenic lineages were identified in MSCs in different culture conditions. MSCs cultured in 10% FBS + 5% HPL medium (M2) displayed the same expression profile for the osteogenic markers CaSR and PTHR as in standard medium (M1) before (D0) and after (D14) differentiation. In contrast, the expression of these proteins was clearly decreased in 10% HPL (M3) and 5% HPL (M4) media. We noted the same results with the adipogenic marker (Leptin) (Figure 5). The expression of VSM differentiation proteins (ASMA and SM22α) seems not to be affected by the type of expansion medium (Figure 5).

### Clonogenic and proliferative capacities of mesenchymal stem cells cultured in various expansion media

#### Colony-forming unit-fibroblast assays

**Figure 6:**
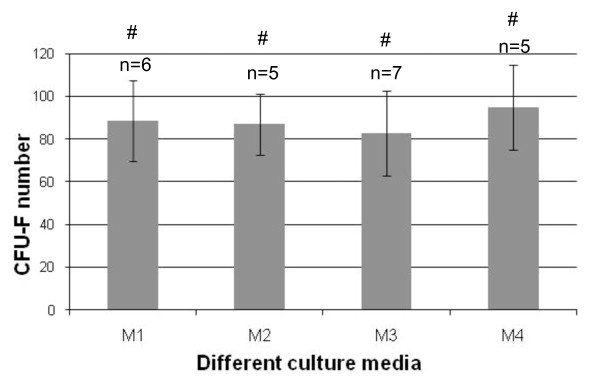
**Effect of expansion media on colony-forming unit-fibroblast (CFU-F) counts per 10^6 ^bone marrow mononuclear cells**. The media are M1 (10% FBS + FGF2), M2 (10% FBS + 5% HPL), M3 (10% HPL), and M4 (5% HPL). Data shown represent mean ± standard error of the mean from the indicated number of experiments. ^#^No significant difference; *P *> 0.05.

MNCs initially seeded in the four expansion media were assayed for CFU-F. No clear difference was observed in colony counts for 10^6 ^BM MNCs plated when comparing the output in FBS- and HPL-supplemented expansion culture media (Figure 6). HPL-supplemented cultures resulted in significantly larger colonies, which appeared densely packed with very small spindle-shaped cells compared with colonies in FBS cultures formed by only loosely connected cells (Figure 3: M2-M3 versus M1). The growth-promoting effect of HPL on CFU-F was associated with increased density of total cells at confluence.

#### Population doubling time

**Figure 7:**
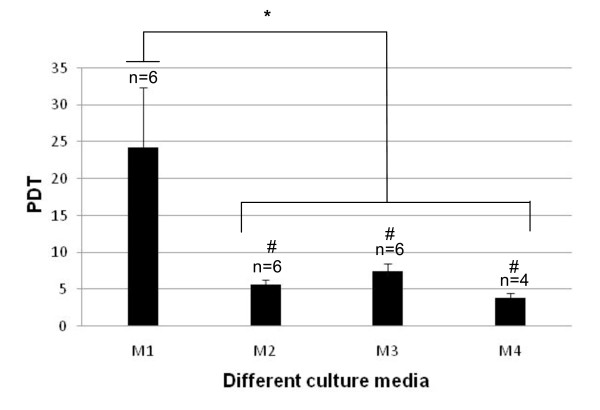
**Population doubling time (PDT) in days of adherent cells cultured in different expansion media between passage 1 (P1) and passage 2 (P2)**. The media are M1 (10% FBS + FGF2), M2 (10% FBS + 5% HPL), M3 (10% HPL), and M4 (5% HPL). Data shown represent mean ± standard error of the mean from the indicated number of experiments. *Significant difference; *P *< 0.0007. ^#^No significant difference; P > 0.05.

Expansion rates of MSCs were very different when cultured in media supplemented with or without HPL (Figure 7). Thus, PDTs of MSCs cultured in regular medium containing FBS with FGF2 (M1) remained longer than in HPL-supplemented cultures (M2: 5.6 ± 0.5 days; M3: 5.8 ± 1.03 days; and M4: 3.8 ± 0.7 days; *P *< 0.0007).

### Cytokine expression profile of mesenchymal stem cells

**Figure 8:**
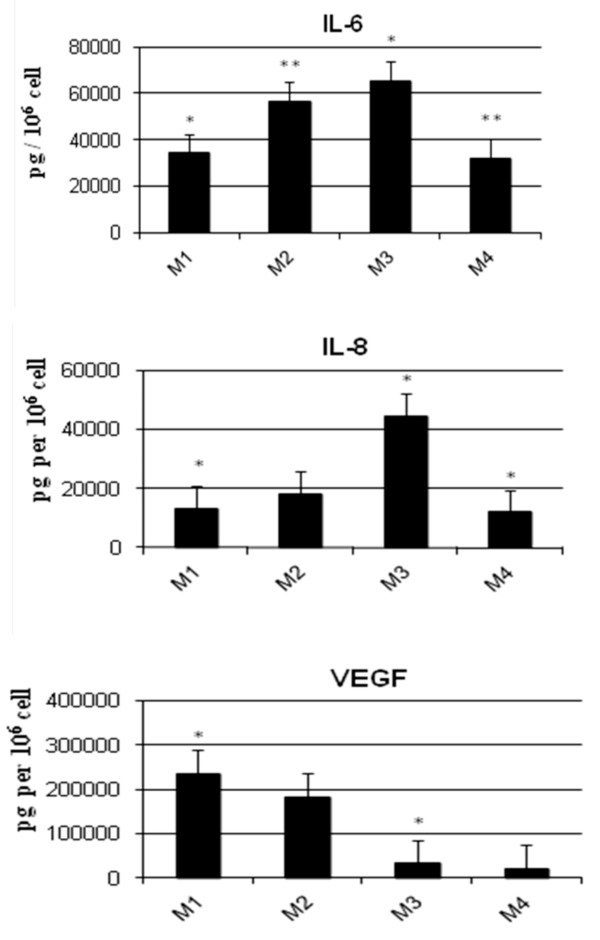
**Cytokine expression profile of mesenchymal stem cells (MSCs) cultured in different expansion media**. Cytokine concentrations (expressed in picograms per 10^6 ^cells) were evaluated in MSC supernatants at passage 2 (P2) after culture in M1 (10% FBS + FGF2) (*n *= 4), M2 (10% FBS + 5% HPL) (*n *= 6), M3 (10% HPL) (*n *= 4), and M4 (5% HPL) (*n *= 3). **(A) **IL-6 (*P = 005; **P = 0.01). **(B) **IL-8 (*P < 0.05). **(C) **VEGF (*P < 0.01). IL, interleukin; VEGF, vascular endothelial growth factor.

Cytokine expression profile of MSCs from BM was compared by using the Bio-Plex^™ ^cytokine assay system (BD Biosciences) in accordance with the instructions of the manufacturer. MSC supernatants were collected after 3 to 4 days of culture at P2 with M1, M2, M3, and M4 for BM. This technique allowed the simultaneous measurement of concentrations of several cytokines that included FGF2, RANTES, VEGF, IL-6, IL-8, G-CSF, and GM-CSF. In parallel to MSC supernatants, background cytokine concentrations were evaluated in each corresponding medium used (M1, M2, M3, and M4). In these growth media, all of the cytokines - except for RANTES, which was present at high concentrations in HPL-containing media (M2, M3 and M4) but was at very low concentrations in standard medium M1 (lacking HPL) - were undetectable. MSC supernatants contained IL-6, IL-8, and VEGF at variable levels according to the different expansion culture conditions. Thus, the presence of HPL appeared to increase IL-6 and IL-8 concentrations (Figure 8A, B), while FGF2, G-CSF, and GM-CSF remained undetectable in all culture conditions.

## Discussion

This study points out the interest in HPL as a replacement for FBS in culture media for expansion of human BM MSCs. Thus, HPL-containing media not only preserve their phenotype as well as their differentiation capacity but also shorten culture time by increasing their growth rate. Nevertheless, some differences exist in terms of cytokines produced, suggesting functional differences between MSCs expanded in media supplemented with HPL and FBS. This has to be considered for particular clinical applications.

The possibility to use animal serum-free culture media has been reported in several recent studies by substituting FBS with human-derived supplements such as HPL or human serum [17-25]. In the present study, MSC expansions were performed in three different HPL-supplemented media consisting of BGM (α-MEM) with (M2) or without (M3 and M4) FBS and compared with the standard medium devoid of HPL (M1). M1 (supplemented with 10% FBS + 1 ng/mL FGF2) represents the reference medium for MSC expansion in our laboratory. In M2, FGF2 was replaced by 5% HPL (vol/vol). FBS-free M3 and M4 media were supplemented with 10% and 5% HPL, respectively. The four media were used for expansion of BM-derived MSCs to study the influence of HPL on MSC functions. Our study clearly showed that the presence of HPL is necessary for MSC growth to substitute the standard expansion medium containing the FBS.

The addition of HPL in expansion media did not modify the immunophenotype of MSCs, irrespectively of the amounts used and of the presence or absence of FBS. These cell display a typical feature of MSCs bearing CD73, CD90, CD105, and CD106 and lacking the hematopoietic markers CD45, CD34, and CD14. Immaturity marker expression did not vary with low levels of CD49a, and undetectable levels of CD133. This lack of consistent immunophenotypic changes of MSCs cultured in HPL-supplemented media has been reported [8,19-21,25,26].

We demonstrated that adherent cells expanded in HPL-supplemented media were multipotent since they were able to differentiate toward four mesenchymal pathways (adipogenic, osteogenic, chondrogenic, and VSM). Thus, we confirm (in addition to VSM pathway) the data of previous studies [19,21-23,27]. However, in M3 containing 10% HPL, MSCs displayed a weak adipogenic differentiation with only a few vesicular adipocytes stained with Nile red.

To assess more precisely the differentiation state of MSCs after expansion in the different media studied and their subsequent differentiation potential, we studied the expression of several differentiation genes by RT-PCR and of proteins by Western blotting for adipogenic, osteogenic, and VSM lineages. These analyses revealed that BM-derived MSCs expanded in all media already express (at both mRNA and protein levels) all of these differentiation markers without any differentiation induction. These data are consistent with those of Delorme and colleagues who demonstrated that non-differentiated human BM-derived MSCs are characterized by lineage priming [28].

However, these markers decreased or even seemed to be absent at D0 (before induction) in the media containing the HPL as a supplement (M2, M3, and M4).

This includes early-appearing genes for adipogenic (*PPARγ *and *LPL*), osteogenic (*RUNX2 *and *ALP*), and VSM (*ASMA*) lineages but also specific proteins of adipogenic (Leptin), osteogenic (CaSR and PTH), and VSM (ASMA and SM22α) lineages. This HPL-induced differentiation defect of MSCs is rather due to an inhibition of adipogenic and osteogenic marker expression than to an expansion of a more immature MSC subpopulation as indicated by immunophenotypic analysis. Nevertheless, this inhibitory effect had no impact on real multipotency of MSCs, since they fully differentiated further toward adipogenic, osteogenic, chondrogenic, and VSM lineages.

To complete these phenotypical and functional studies, we investigated clonogenic and proliferative capacities of MSCs according to the presence or absence of HPL in expansion media. Clonogenic efficiency was evaluated within the MNC fraction initially seeded in the four different media from BM. HPL (alone or combined with FBS) did not affect the recruitment of the clonogenic MSC fraction with values ranging from 80 to 100 CFU-F per 10^6 ^MNCs. This suggests that MSCs grown in such media are functionally close to those obtained in regular media (devoid of HPL). This parallels the lack of immunophenotypic changes for immature populations (CD49a and CD133) known to contain the clonogenic cell population [29,30].

Although HPL-supplemented media did not modify clonogenic efficiency of BM-derived MNCs colonies appeared larger than in regular medium and were formed by small, densely packed, spindle-shaped cells. Such a growth-promoting effect on formed colonies suggests an impact of HPL on the proliferative capacity of MSCs. This was confirmed by calculating the PDT of MSCs between passage 1 and passage 2; the PDT was considerably shortened in all of the media containing HPL. This effect can be attributed to HPL itself since it was not enhanced by the presence of FBS. The only replacement of FBS by 5% HPL in BGM led to a maximal effect with about a fourfold reduction of PDT.

Thus, the use of HPL can be of particular interest in clinical applications to shorten human MSC culture duration and then minimize the risk of entering senescence and transformation [31]. Higher growth-promoting activity of human substitutes for FBS (particularly HPL) on MSCs has been pointed out by several authors [12,18-20,22,24,27,32,33]. Bieback and colleagues [21] showed that only HPL-supplemented medium, in contrast to human sera- and platelet-enriched plasma, is able to increase the growth rate of MSCs cultured in FBS-supplemented medium. The high levels and numbers of platelet-derived growth factors contained in HPL are likely to be responsible for the accelerated growth rate of MSCs that could act in synergy with each other. Many growth factors have been found in platelet concentrates [27,34]. Among these, TGF-β, PDGF, and FGF2 have been shown to be critical for both differentiation and proliferation of MSCs [35].

In addition, we assessed the cytokine secretion profile of MSCs expanded in regular medium (M1) and in three HPL-supplemented media (M2, M3, and M4). We used a multiplex bead-based immunoassay array to simultaneously measure concentrations of seven cytokines (FGF2, RANTES, VEGF, IL-6, IL-8, G-CSF, and GM-CSF) in MSC supernatants in comparison with corresponding media. All MSC supernatants contained IL-6, IL-8, and VEGF at variable levels according to the different expansion culture conditions, whereas FGF2, G-CSF, and GM-CSF remained undetectable in supernatants in all culture conditions of MSCs. The presence of HPL increased IL-6 and IL-8 concentrations, in particular, at 10%. Variations of RANTES expression cannot be evaluated, because of the high level of this chemokine in HPL-supplemented media, whereas consistent baseline levels were found in supernatants of MSCs cultured in regular medium. In fact, the expression cytokine profile in MSCs cultured in regular medium is now well established. We previously reported by mRNA array that MSCs express a number of tyrosine kinase receptor cytokines, including VEGF, Ang-1, PDGFA, FGF1 and 2, EGF, IGF-1, and HGF, but also a number of CC and CXC chemokines, including RANTES, MCP-1, GRO alpha and beta, IL-8, and SDF-1 [36]. Similar results were found in other studies at the protein level by large-scale analysis [37,38]. Although constitutive expression of the hematopoietic growth factors G-CSF and GM-CSF, has also been reported in other studies [37,39], these growth factors were not found in the present study. **{ **This may reflect technical differences with lower sensitivity (but higher specificity) of the Bio-Plex^™ ^technique, as indicated by the lack of detection of cytokines (except for RANTES) in HPL-containing media. Otherwise, we demonstrated here that secretion of both the pro-inflammatory and hematopoietic cytokine IL-6 and the chemokine IL-8 was increased in MSCs previously expanded in HPL-supplemented medium, as described elsewhere for IL-6 [21]. This indicates that HPL contains strong inductors of such cytokines that could minimize the anti-inflammatory activity of MSCs.

## Conclusions

MSCs can be expanded in media supplemented with HPL that can totally replace FBS as well as FGF2. HPL represents an important tool for clinical use to avoid animal-derived additives. HPL does not induce major phenotypical changes. Despite some variations in differentiation markers, MSCs retain their differentiation capacity toward four mesenchymal lineages. The clear growth-promoting activity of HPL can be particularly useful to shorten expansion culture duration of human MSCs to make their clinical-scale production safer.

## Abbreviations

α-MEM: alpha-minimum essential medium; *ALP*: alkaline phosphatase; ASMA: alpha smooth muscle actin; BGM: basic growth medium; BM: bone marrow; CaSR: calcium-sensing receptor; CFU-F: colony-forming unit-fibroblast; D0: day 0; D14: day 14; DMEM-HG: Dulbecco's modified Eagle's medium-high glucose; EDTA: ethylenediaminetetraacetic acid; FBS: fetal bovine serum; FGF2: fibroblast growth factor 2; FITC: fluorescein isothiocyanate; G-CSF: granulocyte colony-stimulating factor; GM-CSF: granulocyte-macrophage colony-stimulating factor; HPL: human platelet lysate; IL: interleukin; *LPL*: lipoprotein lipase; MNC: mononuclear cell; MSC: mesenchymal stem cell; P1: passage 1; P2: passage 2; PBS: phosphate-buffered saline; PDT: population doubling time; PE: phycoerythrin; PerCP: peridinin chlorophyll protein; *PPARγ*: peroxisome proliferator-activated receptor-gamma; PTHR: parathormone receptor; RANTES: regulated on activation normal T cell expressed and secreted; RT-PCR: reverse transcription-polymerase chain reaction; *RUNX2*: Runt-related transcription factor 2; SM22α: transgelin; TGF-β: transforming growth factor-beta; VEGF: vascular endothelial growth factor; VSM: vascular smooth muscle.

## Competing interests

The authors declare that they have no competing interests.
